# Acute effects of inspiratory muscle warm-up on muscular performance and pulmonary function in natural male bodybuilders

**DOI:** 10.1186/s13102-026-01816-4

**Published:** 2026-06-30

**Authors:** Tülay Ceylan, Levent Ceylan, Coşkun Yılmaz, Adela Badau, Hamza Küçük

**Affiliations:** 1 Ministry of National Education, Çorum, Türkiye; 2 Department of Sport Management, Department of Hitit University, Faculty of Sport Sciences, Çorum, 19300 Turkey; 3 Department of Sport Management, Kelkit Aydın Doğan Vocational School, Gümüşhane University, Gümüşhane, 29600 Turkey; 4 Faculty of Physical Education and Mountain Sports, Transylvania University of Brasov, Brasov, 500036 Romania; 5 Department of Physical Education and Sport Teaching, Yasar Dogu Faculty of Sport Sciences, Ondokuz Mayis University, Samsun, 55200 Turkey

**Keywords:** Exercise performance, Acute interventions, Human physiology, Inspiratory muscle warm-up, Bodybuilders, 1RM

## Abstract

**Background:**

Inspiratory muscle warm-up (IWU) has been hypothesized to enhance athletic performance by pre-activating respiratory muscles; however, evidence in professional natural male bodybuilders is scarce. The present study investigated the acute effects of IWU on One-Repetition maximum (1RM) bench press performance and pulmonary function.

**Methods:**

The present randomized controlled experimental study comprised 20 male athletes with an average training experience of 3.21 ± 1.44 years, who trained for more than 5 h per week and had participated in national or international competitions. At the commencement of the study, all participants underwent a series of pulmonary function tests (PFTs), measurements of maximal inspiratory and expiratory pressures (MIP and MEP), and a 1RM test. Following the initial assessments, which were conducted seventy-two hours prior, participants were randomly assigned to either the IWU group (*n* = 10) or the control (CON) group (*n* = 10). While the IWU group performed both inspiratory muscle warm-up and standard exercise warm-up, the CON group performed only the standard warm-up. Subsequent to the execution of the warm-up protocols, both groups were subjected to re-evaluation through the means of the 1RM test and respiratory parameter measurements.

**Results:**

The IWU group demonstrated a 5.96% greater enhancement in 1RM performance in comparison to the control group (*p* < 0.001). Furthermore, significant increases were observed in FVC, FEV1, and FEV1/FVC following IWU, with improvements of 2.57%, 2.44%, and 2.65%, respectively (*p* < 0.001). Furthermore, an additional 2.39% enhancement in MEP was identified in favour of the IWU group (*p* = 0.008). The alterations in MIP and PEF exhibited by the two groups were analogous, with no statistically significant disparities observed (*p* > 0.05).

**Conclusions:**

The present study has demonstrated that IWU has the capacity to rapidly enhance maximal strength and respiratory function in professional bodybuilders. Consequently, IWU can be advocated as an efficacious warm-up technique to augment exercise performance in bodybuilding athletes.

**Trial registration:**

This trial was registered at ClinicalTrials.gov under the title “Acute effects of inspiratory muscle warmup on muscular performance and pulmonary function in natural bodybuilders’’. ClinicalTrials.gov NCT07390149, Date 18012026. (Retrospectively)

**Supplementary Information:**

The online version contains supplementary material available at https://doi.org/10.1186/s13102-026-01816-4.

## Introduction

Resistance training constitutes a fundamental component of numerous bodybuilders’ training regimens, particularly those whose objective is to augment muscle mass. These athletes typically engage in high-resistance strength training a minimum of three times per week to promote muscle hypertrophy [1], in conjunction with exercise modalities that involuntarily activate the respiratory muscles. The Valsalva maneuver, frequently observed during high-intensity resistance exercise, plays a key role in trunk stabilization by increasing intra-abdominal pressure; however, it simultaneously elevates cardiovascular and respiratory strain. This dual effect highlights the importance of respiratory muscle preparedness prior to maximal efforts [2, 3]. The breathing pattern that is induced during weightlifting provides a stimulus that is similar to that of inspiratory muscle training (IMT), thereby contributing to the strengthening of these muscle groups [3]. The functionality of the respiratory system is contingent on the capacity of the respiratory muscles [4], and chronic or acute interventions targeting these muscles can directly affect both respiratory and exercise performance [5].

Exercises aimed at enhancing the contractile strength of respiratory muscles have been shown to improve pulmonary functional parameters such as maximal inspiratory pressure (MIP), maximal expiratory pressure (MEP), vital capacity (VC), and total lung capacity (TLC) [6, 7]. Consequently, this can result in improved athletic performance. The respiratory muscles are also actively engaged during the Valsalva manoeuvre (VM), a technique frequently used in strength training. This technique, when combined with the diaphragm and the muscles of the abdomen, performs a pivotal role in the stabilization of the trunk and the generation of intra-abdominal pressure [8, 9]. It has been demonstrated that these physiological adaptations, which underpin core stability, concomitantly serve to provide strengthening stimuli for the respiratory muscles. Furthermore, these adaptations have been demonstrated to be positively correlated with enhancements in maximal lifting performance.

Recent studies have demonstrated that IWU protocols can enhance the functional capacity of respiratory muscles by improving oxygenation and fatigue tolerance [10, 11]. As postulated by Tong and Fu, Özdal and Bostanci, and Yılmaz et al., IWU has been demonstrated to exert a favourable influence on the efficacy of physical exertion, concomitantly engendering a reduction in the perception of dyspnea [12–14]. However, the extant literature offers only limited evidence regarding the effects of IMT on single repetition maximum (1RM) performance, which is related to maximal power output [15]. Nevertheless, a consensus on the acute effect of IWU on 1RM performance remains elusive.

Warm-up protocols are widely employed strategies used to prepare the body for competition [16]. However, beyond conventional warm-up routines, specific preparatory exercises targeting the inspiratory muscles have demonstrated the potential to enhance both respiratory muscle endurance and optimize One-Repetition maximum (1RM) performance, particularly in bodybuilders exposed to high-intensity training loads at the professional level. In this context, this study aimed to examine the acute effects of inspiratory muscle warm-up on maximal strength performance and respiratory function in trained male natural bodybuilders using a randomized parallel-group design. It was hypothesized that inspiratory muscle warm-up would lead to greater improvements in 1RM performance and respiratory parameters compared to a control condition.

## Materials and methods

### Participants

Twenty male athletes who had competed in national and international professional natural bodybuilding competitions were recruited for this study. All participants were competitive male natural bodybuilders with experience at both national and international levels. The distribution was consistent across groups, with all athletes in the IWU and CON groups having competed in national and international events, indicating a homogeneous and high-performance sample. The participants had an average experience in natural bodybuilding of 3.21 years, with a standard deviation of ± 1.44 years, and trained for more than five hours per week. The data collection period commenced in July 2025.

**Fig. 1:**
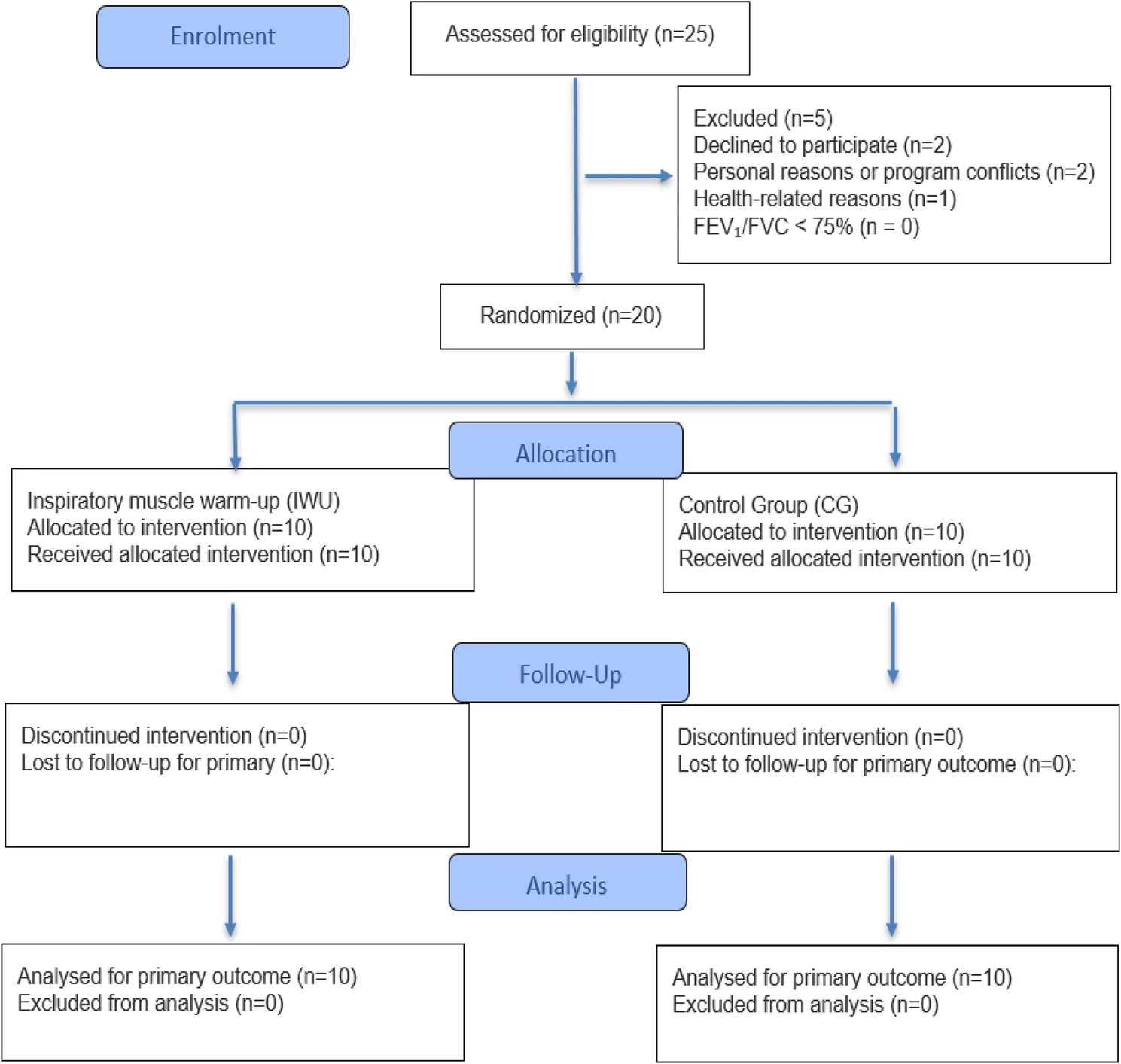
CONSORT flow diagram of participant recruitment, allocation, follow-up, and analysis

This study was designed as a randomized controlled trial with a parallel-group design and repeated measures. Participants were randomly assigned to one of two independent groups (IWU or CON) and remained in their allocated group throughout the study. No crossover or condition switching was implemented. The repeated assessments across visits were conducted to evaluate acute responses over time and should not be interpreted as a crossover design. Participants attended three laboratory sessions for baseline assessment and post-intervention measurements; however, each participant completed only their assigned intervention condition. The overall study design and participant flow are presented in Fig. 1. The study design and reporting follow CONSORT guidelines for parallel-group randomized trials. An a priori sample size estimation was conducted using G*Power (v3.1) based on a repeated-measures ANOVA (within–between interaction). With an assumed large effect size (f = 0.40), α = 0.05, power = 0.80, two groups, and two repeated measurements, the required total sample size was estimated to be approximately 20 participants. This estimation was based on prior literature reporting substantial effects of inspiratory muscle interventions on performance outcomes [15]. The numbers from 1 to 20 were randomly assigned to two groups by a computerized program in order to ascertain which group the subjects forming the sample would be included in (https://www.randomizer.org/). In an effort to eliminate the potential effects of hand dominance and strength, the study exclusively included right-hand dominant individuals [17]. Furthermore, all participants underwent identical procedures in order to control for potential contralateral effects [18]. Individuals failing to meet the following criteria were excluded from the study.

The following criteria were used to select participants for the study: The inclusion criteria stipulated that individuals would be considered eligible if they met the following criteria: a) They were professional natural bodybuilders with more than three years of experience. Individuals were deemed ineligible on the basis of a medical history that included any chronic or acute respiratory disease. (b) Individuals were considered ineligible if they were taking any prescription medication that could affect their response to exercise. (c) In accordance with the stipulated criteria, individuals were deemed to be ineligible for participation in the study if they had a history of current smoking or had smoked within the past year. (d) Individuals were considered ineligible for participation in the study if they had participated in similar studies within the previous six months. Prior to participation, all individuals were required to sign a formal waiver, thereby affirming their adherence to the World Anti-Doping Agency Code. Prior to the initiation of the study, all participants were requested to provide both verbal and written consent.

### Training background

The RT protocols applied by the athletes participating in the study were developed in accordance with the National Strength and Conditioning Association (NSCA)’s recommendations for optimal athletic development. Training programs for both groups were organized by an experienced senior coach. The training was conducted 6 days per week, with a maximum interval of 45 s for each exercise and a 30-s inter-set break time. A 5–10-min warm-up routine, comprising a variety of dynamic movements, was conducted prior to each training session. All participants followed a structured resistance training program typical of competitive natural bodybuilders (Çelikel et al., 2025). Training was performed 6 days per week, using a split routine targeting different muscle groups across sessions (e.g., chest–triceps, back–biceps, legs, shoulders). Exercises primarily consisted of multi-joint and single-joint movements (e.g., squats, bench press, deadlift, rows, and isolation exercises). Training intensity generally ranged between 70 and 90% of 1RM, with 3 sets per exercise and 8–12 repetitions per set, consistent with hypertrophy- and strength-oriented training. Rest intervals between sets were typically 60–120 s, depending on the exercise and load.

### Data collection

The data collection period occurred between the 1st and 6th of July 2025. Prior to their involvement in the study, all subjects provided both verbal and written informed consent. Participants attended three laboratory sessions. During the first visit, all baseline measurements (PFT, MIP/MEP, and 1RM) were obtained prior to randomization. Following baseline assessment, participants were randomly assigned to either the IWU or CON group. The second visit was conducted to obtain pre-intervention measurements without any warm-up protocol and to ensure measurement reliability. During the third visit, participants performed their assigned condition (IWU + general warm-up or general warm-up only), followed by post-intervention assessments [14]. No participant was exposed to both conditions. Each group comprised ten professional male natural bodybuilders. PFT and MIP-MEP tests were reassessed, and 1RM performance was measured immediately following the IWU intervention. In order to control for circadian variations, all laboratory visits were conducted at the same time of day (between 09:00 and 12:00). Participants were notified 72 h in advance of each visit and were instructed to refrain from high-intensity physical activity during that period. It was determined that a recovery period of 72 h should be observed between each testing session.

### Body composition measurement

The Gaia 359 Plus Body-pass bioelectrical impedance analyzer was employed to evaluate the body composition of athletes who presented themselves at the laboratory of Gümüşhane University. The device employs a measurement technique that generates and calculates information about the tissue based on the type of resistance encountered by low electrical currents as they pass between body tissues. The height, body weight, and body mass index (BMI) of the subjects were determined by means of the use of the Gaia 359 Plus BodyPass. The subjects were instructed to stand on the analyzer with their entire soles in contact and to remove all outer clothing, including t-shirts and shorts. To ensure measurement reliability, participants reported to the laboratory in a fasted state (≥ 8 h) and were instructed to avoid vigorous physical activity, alcohol, and caffeine for at least 24 h prior to testing. Prior to the commencement of the measurement, subjects were instructed to remove all metal objects from their bodies. Following a thorough examination of the group averages of the study participants, it was determined that the IWU group had a mean age of 23.10 ± 2.56 years, a body weight of 84.35 ± 6.25 kg, and a height of 180.9 ± 4.89 cm. In comparison, the control group exhibited a mean age of 22.78 years (± 1.39 years), a body weight of 83.33 kg (± 3.20 kg), and a height of 179.44 centimeters (± 4.07 centimeters). There were no significant differences between the IWU and Control groups regarding age, height, body weight, or BMI (*p* > 0.05), indicating that the groups were homogeneous at baseline (Table 1).

**Table 1:** Descriptive characteristics of the IWU and control groups

Descriptive	IWU (*n*:10)		Control (*n*:10)		*p*
	Mean	SD	Mean	SD	
Height (cm)	180.90	4.89	179.44	4.07	0.493
Body weight (kg)	84.35	6.25	83.33	3.20	0.667
BMI (kg/m^2^)	24.77	1.35	23.73	0.63	0.089
Age (years)	23.10	2.56	22.78	1.39	0.742

### Pulmonary function tests (PFTs)

Pulmonary function parameters, including peak expiratory flow (PEF), forced expiratory volume in one second (FEV1), the FEV1/FVC ratio (Tiffeneau index), and forced vital capacity (FVC), the total volume of air exhaled forcefully after a maximal deep inhalation, were measured using a MGF Diagnostics CPFS/D USB spirometer. Each participant was fitted with a nose clip and given instructions to inhale deeply and exhale forcefully and rapidly through the mouthpiece connected to the spirometer. Three trials were recorded, and the highest value was used for analysis. The intraclass correlation coefficient (ICC) was calculated as PEFmax (0.813), FEV1 (0.818), FEV1/FVC (0.841), and FVC (0.831). Participants with an FEV1/FVC ratio below 75%, as well as those with a history of pulmonary disease, upper respiratory tract infection, or existing voice disorders, were excluded from the study. No participants were excluded based on the FEV₁/FVC criterion, as all screened individuals presented values ≥ 75%. PFTs were conducted in accordance with the 2002 guidelines of the American Thoracic Society and the European Respiratory Society (ATS/ERS) [19]. Verbal encouragement was provided to ensure maximum effort during all respiratory tests [15].

### Maximal inspiratory (MIP) and expiratory (MEP) pressure measurements

MIP and MEP were measured using a portable, handheld mouth pressure monitor (MicroRPM; CareFusion Micro Medical, Kent, UK) in accordance with the 2002 guidelines of the American Thoracic Society and the European Respiratory Society (ATS/ERS) [19]. Following the acquisition of the necessary equipment, specifically the filters and mouthpieces, the nasal airway was occluded using a nose clip. MIP measurements were initiated from residual volume, while MEP measurements were initiated from total lung capacity. The procedure was repeated until the difference between the two best efforts was within 5%, and the average value was recorded in cmH2O [14]. The intraclass correlation coefficient (ICC) was calculated as MIP (0.829) and MEP (0.862). This test was performed for the IWU protocol, as the resistance of the POWERbreathe device is to be adjusted according to each participant’s maximal inspiratory pressure (MIP 40%). All participants demonstrated MIP and MEP values within established normative ranges for healthy adult males, according to previously published reference values. Accordingly, no participants with clinically reduced respiratory muscle strength were included.

### Inspiratory muscle warm-up (IWU)

The POWERbreathe device (IMT Technologies Ltd., Birmingham, UK) was utilized for IWU. In the context of the IWU protocol, the resistance of the POWERbreathe device was calibrated to 40% of the participant’s maximal inspiratory pressure (MIP). Participants were instructed to perform two sets of 30 breaths (inspiratory and expiratory) with a 60-second rest interval between sets [20]. All participants in the IWU group performed the inspiratory muscle warm-up immediately prior to the strength testing.

### One-repetition maximum (1RM) estimation method (bench press)

In recent years, there has been an increasing utilization of predictive equations in order to overcome the limitations associated with the direct assessment of One-Repetition maximum (1RM). The majority of these models are predicated on the principle that loading within the range of 2 to 10 repetitions yields the most accurate estimations [21, 22]. In accordance with this, the 1RM test was employed in the present study for the purpose of maximal strength assessment, and the Mayhew equation was utilized for prediction calculations due to its lower absolute error rate [23]. Prior to the 1RM test, the initial load was determined on the basis of each participant’s training history, as a weight that they could perform for a minimum of two repetitions, or believed they could successfully lift. The bench press (BP) exercise was selected for maximal strength assessment because it reflects the functional relationship between the respiratory system and upper extremity musculature [24].

During the bench press procedure, participants were positioned on a flat bench with a 90° elbow angle and a 45° arm-to-torso angle. The width of the grip was standardized in accordance with the guidelines established by the International Powerlifting Federation (IPF). It is important to note that all repetitions were performed at a controlled tempo, which was defined as V/0/V/0 (voluntary eccentric and concentric phases) [25, 26]. The utilization of wrist straps was not authorized. Verbal encouragement and feedback were provided throughout the 1RM attempts to ensure maximal effort [27]. The load (in kilograms) and the number of repetitions were then used to estimate the 1RM value via the Mayhew equation.

$$\text{Mayhew Formula:}\:\mathrm{1RM}=\left(\mathrm{load}/52.5+41.9\cdot\mathrm{e}^{\wedge}\left(-0.055\:\times\mathrm{repetitions}\right)\right)/{100}$$

### Statistical analysis

Statistical analyses were performed using SPSS (Version 27.0 for Windows, Chicago, IL, USA) software, with a statistical significance level set at 0.05. The Shapiro‒Wilk normality test was performed in order to ascertain the homogeneity of the sample. Repeated measures two-way analysis of variance and Bonferroni correction were used to analyze differences in 1RM, respiratory function, and respiratory muscle strength measurements between trials. Furthermore, the effect size in pairwise group comparisons was calculated using partial eta-squared (ηp^2^). The interpretation of the parameter ηp^2^ is as follows: small values, such as 0.01, indicate a small effect size; medium values, such as 0.06, indicate a medium-sized effect; and large values, such as 0.14, indicate a strong effect [28]. The intraclass correlation coefficient (ICC) was calculated in order to assess the reproducibility of measurements obtained from the same observer. The following interpretation of the results is proposed: scores between 0.50 and 0.75 are considered moderate, scores between 0.75 and 0.90 are considered good, and scores of 0.90 and above are considered excellent [29].

## Results

Table 2 presents the pre- and post-intervention comparisons of maximal strength, pulmonary function, and respiratory muscle strength parameters in the IWU and control groups.

**Table 2:** Comparison of pre- and post-training values

Parameters	Group	Pre-test Mean X̅±SD	Post-test Mean X̅±SD	Δ%	Time Effect		Group x Time Interaction	
					F	*p* (ηp^2^)	F	*p* (ηp^2^)
1RM	IWU	97.95 ± 8.66	103.98 ± 8.80	6.15	160.480	*p* < 0.001 (0.904)	141.560	*p* < 0.001 (0.893)
	CG	96.11 ± 8.01	96.30 ± 7.71	0.19				
FVC	IWU	4.67 ± 0.32	4.81 ± 0.34	3	43.180	*p* < 0.001 (0.718)	24.972	*p* < 0.001 (0.595)
	CG	4.60 ± 0.26	4.62 ± 0.26	0.43				
FEV₁	IWU	4.9 ± 0.26	5.05 ± 0.25	3.06	118.279	*p* < 0.001 (0.874)	51.077	*p* < 0.001 (0.750)
	CG	4.79 ± 0.26	4.82 ± 0.26	0.62				
FEV₁ /FVC	IWU	89.30 ± 2.36	92 ± 1.25	3.02	20.524	*p* < 0.001 (0.547)	12.494	0.003 (0.424)
	CG	88.78 ± 2.05	89.11 ± 2.09	0.37				
PEF	IWU	8.01 ± 0.91	8.43 ± 0.86	5.24	45.698	*p* < 0.001 (0.729)	0.064	0.803 (0.004)
	CG	7.82 ± 0.61	8.21 ± 0.59	4.99				
MIP	IWU	126.3 ± 11.57	135.4 ± 1.64	7.2	180.099	*p* < 0.001 (0.914)	0.598	0.450 (0.034)
	CG	128.44 ± 9.13	136.56 ± 9.79	6.32				
MEP	IWU	139.7 ± 19.1	151.9 ± 19.61	8.73	422.853	*p* < 0.001 (0.961)	8.883	0.008 (0.343)
	CG	143.67 ± 16.16	152.78 ± 17.5	6.34				

**Fig. 2:**
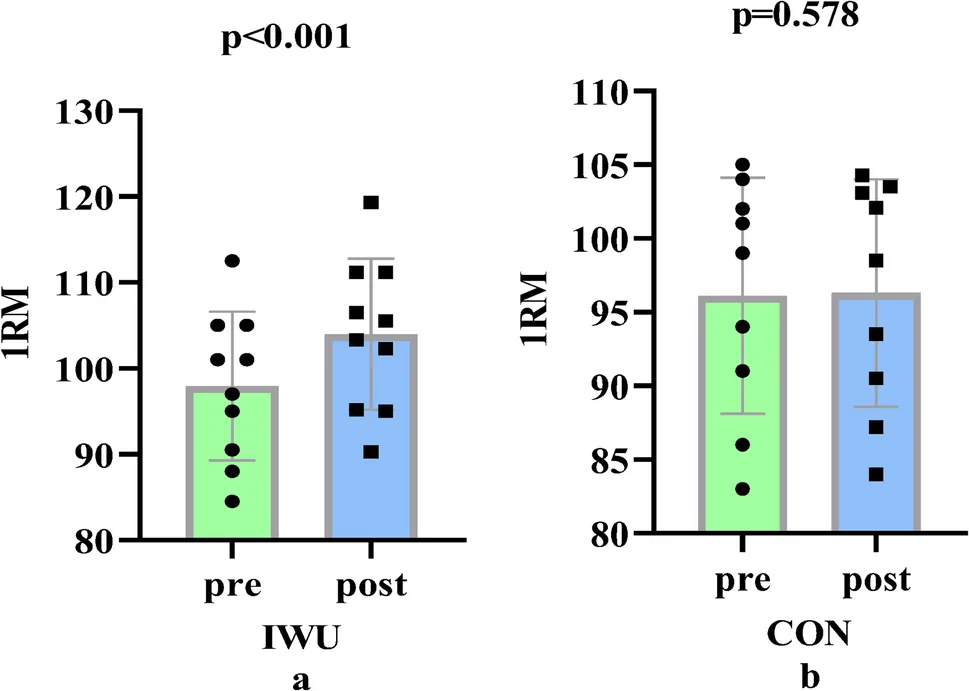
Changes in estimated 1RM performance across time in the IWU and CON groups

Regarding maximal strength performance (1RM), a significant group × time interaction was observed (F = 141.560, *p* < 0.001, ηp² = 0.893), indicating that the magnitude of change differed significantly between groups. Post hoc analyses demonstrated a significant within-group increase in the IWU group (+ 6.15%, *p* < 0.001), whereas no significant change was detected in the control group (+ 0.19%, *p* = 0.578) (Table 2, Fig. 2).

**Fig. 3:**
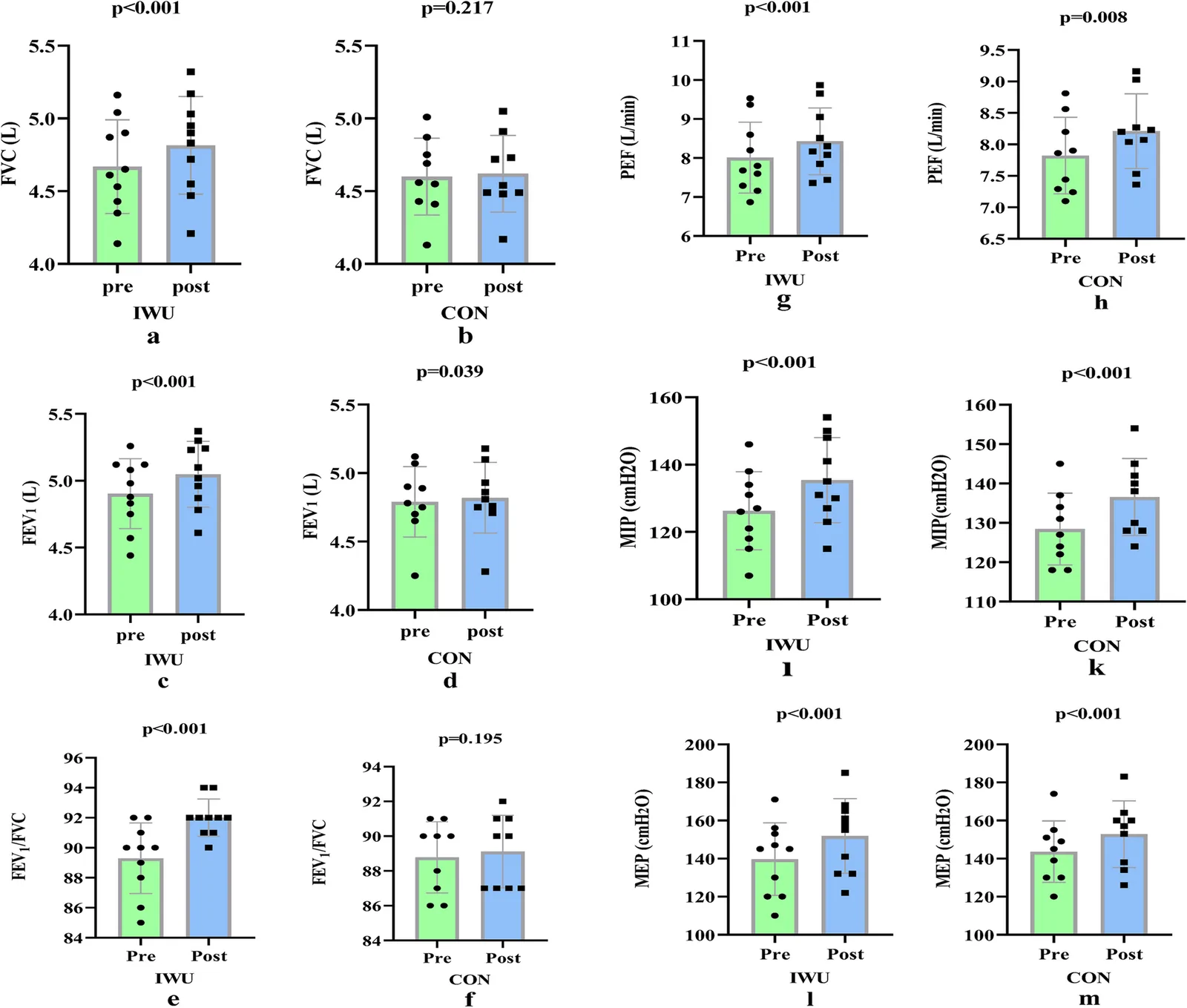
Comparison of respiratory function and respiratory muscle strength. Panels (**a**–**b**) represent forced vital capacity (FVC), panels (**c**–**d**) forced expiratory volume in one second (FEV1), panels (**e**–**f**) the FEV1/FVC ratio, panels (**g**–**h**) peak expiratory flow (PEF), panels (**i**–**k**) maximal inspiratory pressure (MIP), and panels (**l**–**m**) maximal expiratory pressure (MEP). Data are presented for the IWU and CON groups before and after the intervention

For pulmonary function parameters, significant group × time interactions were identified for FVC (F = 24.972, *p* < 0.001, ηp² = 0.595), FEV₁ (F = 51.077, *p* < 0.001, ηp² = 0.750), and FEV₁/FVC (F = 12.494, *p* = 0.003, ηp² = 0.424). The IWU group demonstrated greater improvements in FVC (+ 3.00%), FEV₁ (+ 3.06%), and FEV₁/FVC (+ 3.02%) compared with the control group (+ 0.43%, + 0.62%, and + 0.37%, respectively). The significant interaction effects indicate that these improvements were attributable to the inspiratory muscle warm-up intervention rather than to repeated testing or time-related effects alone (Table 2, Fig. 3a-f).

In contrast, although PEF values increased significantly over time in both groups (IWU: +5.24%; CON: +4.99%), the group × time interaction was not significant (F = 0.064, *p* = 0.803, ηp² = 0.004). Therefore, while both groups exhibited within-group improvements, there is no evidence that the IWU protocol provided a superior benefit compared with the control condition for PEF (Table 2, Fig. 3g-h).

Similarly, significant within-group improvements were observed for MIP in both groups (IWU: +7.20%; CON: +6.32%), accompanied by a significant main effect of time (F = 180.099, *p* < 0.001, ηp² = 0.914). However, the absence of a significant group × time interaction (F = 0.598, *p* = 0.450, ηp² = 0.034) indicates that the magnitude of improvement was comparable between groups. Consequently, no superiority of the IWU intervention can be inferred for MIP (Table 2, Fig. 3ı-k).

Regarding MEP values, the IWU group exhibited an 8.73% improvement (*p* < 0.001), which was 2.39% greater than the control group (6.34%, *p* < 0.001). For MEP, both a significant time effect (*p* < 0.001; ηp² = 0.961) and a group × time interaction (*p* = 0.008; ηp² = 0.343) were observed. Although statistically significant, the magnitude of the interaction suggests a moderate effect (Table 2, Fig. 3l-m).

## Discussion

The findings of this study demonstrate that IWU has significant and beneficial short-term effects on strength performance and respiratory function in professional natural bodybuilders. The main finding of this study is that IWU resulted in a modest improvement in estimated maximal strength (~ 6%), whereas no meaningful change was observed in the control condition. This increase is significant not only in terms of force production but also because it highlights the contribution of inspiratory muscle activation to performance, thereby contributing to the existing literature. The findings suggest that IWU results in significant enhancements in muscular strength performance and respiratory parameters. The observed improvements may be associated with enhanced inspiratory muscle activation and reduced respiratory muscle fatigue. One potential explanation involves alterations in respiratory muscle afferent feedback and respiratory metaboreflex activity, which have been proposed to influence neuromuscular performance in previous studies. However, because these mechanisms were not directly assessed in the present study, such interpretations should be considered speculative and require confirmation in future investigations employing direct physiological measurements. The relatively homogeneous and structured training background of the participants should be considered when interpreting the findings, as resistance training experience, intensity, and volume may influence both respiratory muscle function and strength performance outcomes.

Although the Mayhew equation used in our study provides a practical and widely used method for estimating 1RM, it may introduce additional measurement error compared to direct 1RM assessment. Therefore, the observed ~ 6% improvement should be interpreted with caution, as part of this change may fall within the inherent variability of estimation-based methods [30]. Moreover, while crossover designs may provide greater sensitivity in detecting acute effects, the present parallel-group design reduces potential carryover and fatigue effects associated with repeated maximal testing. The present study extends the existing literature by examining IWU effects in resistance-trained natural bodybuilders, a population that has been underrepresented in previous research.

Recent bodybuilding studies show that it is not one-off inspiratory muscle warm-ups but regular inspiratory muscle training that increases bench press strength, respiratory capacity, and diaphragm thickness in professional natural bodybuilders. Acute inspiratory muscle warm-up performed at approximately 40–60% of MIP improves strength, power measurements, and high-intensity performance in other athletes; however, specific studies on this topic have not yet been conducted in bodybuilders. The closest available evidence in bodybuilders is a 6-week IMT study; acute warming effects, however, have been documented in other sports.

A 6-week IMT program (PowerBreathe at 40% MIP in addition to normal preparation) administered to professional natural bodybuilders increased the bench press 1RM more than the control group (14.39% vs. 9.43%), improved spirometric indices (FVC, FEV_1_, PEF), MIP/MEP, and various muscle parameters, and reduced perceived exertion on the Borg scale. The authors concluded that the addition of progressive IMT (inspiratory muscle training) to competition preparation improves respiratory function, respiratory muscle strength, maximum power, and muscle development in this population [15]. A single IWU session was not tested in this study; the effects are not acute but training-related.

In accordance with extant literature, the present study reports that IWU enhances performance and fatigue resistance across a range of sports disciplines, including competitive events, intermittent running, sprinting, endurance performance, anaerobic power output, and resistance training [31–35]. The mechanism by which IWU exerts its effects appears to be through the pre-activation of the respiratory muscles.

Conversely, some studies have reported no significant effect of IWU on strength outcomes. These discrepancies may be related to differences in timing between warm-up and performance testing, which has been shown to influence the persistence of respiratory-related ergogenic effects [36].

Our findings are partially consistent with previous studies reporting acute performance benefits following inspiratory muscle warm-up. However, the magnitude of improvement observed in the present study (~ 6%) is smaller than that reported in some athletic populations, which may be due to differences in testing protocols, training status, and outcome measures (estimated vs. directly measured 1RM).

The observed increases in MIP and MEP are significant indicators of respiratory muscle strength following IWU. It is hypothesized that these increases may have contributed to enhanced force production through increased intra-abdominal pressure and improved trunk stabilization. As demonstrated in the study [37], there is a demonstrable correlation between lung function and respiratory muscle strength on the one hand, and the endurance of core muscles responsible for maintaining trunk stability on the other. Pre-activation of the respiratory muscles may have facilitated more efficient musculoskeletal integration during the 1RM test by optimizing breath control.

The respiratory function data further corroborate the efficacy of IWU as a preparatory technique, particularly prior to high-intensity exercise. When administered prior to strenuous physical exertion, IWU demonstrated a substantial enhancement in critical respiratory parameters, including FVC, FEV_1_, and the FEV_1_/FVC ratio. This phenomenon can be attributed to an enhancement in the respiratory system’s capacity to withstand demanding breathing maneuvers. The considerable effect size observed for the FEV_1_/FVC ratio (ηp^2^= 0.424) serves to emphasize the efficacy of IWU in enhancing ventilatory capacity. Although analogous enhancements were evident in PEF and MIP values in both the IWU and control groups, the more pronounced enhancements in MEP in the IWU group suggest a potential indirect effect of inspiratory muscle warm-up on expiratory muscle performance.

Moreover, the absence of a statistically significant disparity between the groups in terms of PEF and MIP values prompts the question of whether IWU exerts a selective effect on expiratory muscles or on general ventilatory capacity. A substantial body of research has been conducted on the effects of IWU on sporting performance, with a particular focus on its impact on ventilatory capacity. A substantial body of research has been conducted on this subject, and the findings have repeatedly demonstrated that IWU significantly enhances ventilatory capacity [11, 31, 38–42].

Lomax et al. reported that IWU increased MIP by 11% and enhanced running distance by 5–7% [11]. As demonstrated by Wilson et al., the combination of inspiratory muscle exercises with a conventional swimming warm-up regimen resulted in a substantial enhancement in performance for elite swimmers [38]. As Özdal et al. reported, the implementation of respiratory warm-up exercises led to significant enhancements in anaerobic power (peak power) and a reduction in the time taken to reach peak power [39]. Barnes and Ludge (2021) observed that IMWU produced a modestly positive effect (~ 21 s, 2.8%) on 3200 m running performance in comparison with a standard warm-up [30]. As demonstrated by Manchado-Gobatto et al., the pre-activation of the inspiratory muscles at 40% of maximal inspiratory pressure resulted in enhanced running power and recovery [40]. Cirino et al., concluded that the majority of the analyzed IMWU protocols had a positive effect on inspiratory and performance parameters, likely due to the contractile and biochemical properties of the inspiratory muscles [36].

From a practical perspective, IWU may be considered as a preparatory strategy prior to maximal strength testing or resistance training sessions. However, given the time-sensitive nature of its effects, practitioners should ensure that performance tasks are initiated shortly after the warm-up. Although the present findings suggest potential performance benefits associated with inspiratory muscle warm-up, the results should be interpreted with caution. Replication in larger samples and across different athletic populations is necessary before definitive conclusions can be drawn regarding its effectiveness as a performance-enhancing strategy in professional bodybuilding.

The present study is subject to several limitations. Firstly, the sample consisted exclusively of professional, high-performance, natural male bodybuilders (*n* = 20), which limits the generalizability of the findings to female or amateur athletes. Therefore, the findings cannot be generalized to female populations. It is important to note that small sample sizes may lead to inflated effect size estimates and increase the likelihood of Type I error. Therefore, the observed large effect sizes should be interpreted with caution until replicated in larger samples. The exclusion of athletes who did not participate in national or international competitions resulted in a further restriction of sample diversity. Additionally, muscle strength was assessed using the predicted Mayhew 1RM formula rather than direct 1RM testing, and no supplementary physiological measurements (e.g., diaphragm ultrasound, electromyography, blood lactate, oxygen saturation) were performed to support the mechanistic interpretation of IWU effects. Another limitation is the potential mismatch between the duration of the IWU effect and the time required to complete the 1RM testing protocol. Recent evidence suggests that the physiological effects of inspiratory muscle warm-up may be transient (~ 15 min). Although IWU was performed immediately before testing, the total duration of the 1RM assessment, including multiple attempts and rest intervals, may have exceeded this time window and introduced variability in the magnitude of the effect. Although repeated baseline assessments were incorporated to improve measurement reliability and minimize learning effects, residual familiarization cannot be completely ruled out. Repeated exposure to spirometry, respiratory pressure measurements, and estimated 1RM testing may have enhanced participants’ ability to perform these assessments more effectively over time. This possibility may be particularly relevant for variables such as MIP and PEF, where both groups demonstrated comparable improvements despite the absence of a significant group × time interaction. Consequently, a portion of the observed changes may reflect increased familiarity with the testing procedures rather than a true intervention-specific effect. Future studies may benefit from additional familiarization sessions before baseline testing to further reduce this potential source of bias. Another limitation is that the physiological mechanisms underlying the observed performance improvements were not directly evaluated. Consequently, potential explanations involving respiratory metaboreflex modulation, altered muscle oxygenation, or reductions in respiratory muscle fatigue remain hypothetical and should be interpreted with caution. The study concentrated exclusively on acute responses, neglecting to evaluate chronic adaptations or the sustainability of performance outcomes. The aforementioned factors collectively serve to narrow the scope of the conclusions. It is recommended that future studies be conducted using larger and more diverse sample groups, by making gender-based comparisons, by examining different IWU intensities, and by incorporating advanced physiological assessments. Furthermore, to better isolate the acute effects of inspiratory muscle warming on strength performance, larger sample groups, direct 1RM assessment, and standardized timing protocols should be employed. This will facilitate a more comprehensive understanding of the effects of IWU.

## Conclusions

The primary hypothesis of this study, namely, that IWU would lead to an increase in the pressure-generating capacity of the inspiratory muscles and consequently result in improved 1RM performance, was confirmed. The findings demonstrate that IWU elicits significant acute improvements in both 1RM strength performance and respiratory function in professional natural bodybuilders. Consequently, IWU may represent a promising preparatory strategy for improving acute strength performance and selected respiratory outcomes in professional natural bodybuilders. However, given the relatively small sample size and the specific characteristics of the study population, these findings should be interpreted cautiously until confirmed by larger randomized studies. The results of the present study suggest that IWU may serve as a practical tool for the optimization of performance during resistance training, with the potential to enhance fatigue resistance and support respiratory efficiency. IWU may represent a promising preparatory strategy; however, confirmation in larger randomized trials is warranted. It is recommended that future research concentrate on the evaluation of the effects of varying IWU protocols in terms of duration and intensity on performance outcomes.

## Supplementary Information

Supplementary Material 1.

## Data Availability

The data used in this study can be accessed at [https://doi.org/10.6084/m9.figshare.31663924](https://doi.org/10.6084/m9.figshare.31663924).

## Abbreviations

IWU: Inspiratory muscle warm-up
FVC: Forced vital capacity
FEV1: Forced expiratory volume in one second
FEV_1_/FVC: FEV1/FVC ratio (Tiffeneau index)
PEF: Peak expiratory flow
MIP: Maximal Inspiratory Pressure
MEP: Maximal Expiratory Pressure
1RM: One-Repetition maximum
