# Supplementary material for: Acute effects of inspiratory muscle warm-up on muscular performance and pulmonary function in natural male bodybuilders
Source: BMC Sports Sci Med Rehabil. 2026 Jun 30;18:428. doi: 10.1186/s13102-026-01816-4 (PMC13587494; doi:10.1186/s13102-026-01816-4)
Supplement: Supplementary file 1 [file 13102_2026_1816_MOESM1_ESM.pdf]

CONSORT 2010 Checklist of Information to Include When Reporting a Randomized Trial

Manuscript: Acute effects of inspiratory muscle warm-up on muscular performance and pulmonary function in natural bodybuilders

Section/Topic	Item No	Checklist item	Reported on page No
Title and Abstract	1a	Identification as a randomized trial in the title	1
Title and Abstract	1b	Structured summary of trial design, methods, results, and conclusions	1
Introduction	2a	Scientific background and explanation of rationale	2–3
Introduction	2b	Specific objectives or hypotheses	3
Methods	3	Description of trial design (e.g., parallel, allocation ratio)	4
Methods	4a	Eligibility criteria for participants	4–5
Methods	4b	Settings and locations where the data were collected	5
Methods	5	The interventions for each group with sufficient details to allow replication	6–7
Methods	6	Completely defined pre-specified primary and secondary outcome measures	6–7
Methods	7	How sample size was determined	5
Methods	8a	Method used to generate the random allocation sequence	5
Methods	8b	Type of randomization; details of any restriction	5
Methods	9	Mechanism used to implement the random allocation sequence	5
Methods	10	Who generated the allocation sequence, who enrolled participants, and who assigned participants	5
Methods	11	Similarity of interventions	6
Methods	12a	Statistical methods used to compare groups	8
Methods	12b	Methods for additional analyses	8
Results	13a	Participant flow (numbers assigned, treated, analyzed)	Figure 1
Results	13b	Losses and exclusions after randomization	Figure 1
Results	14	Dates defining the periods of recruitment and follow-up	5
Results	15	Baseline demographic and clinical characteristics	Table 1
Results	16	Number of participants included in each analysis	9
Results	17	Results for each outcome with estimated effect size and precision	Table 2
Results	18	Results of any other analyses performed	9–10
Results	19	All important harms or unintended effects	Not reported
Discussion	20	Trial limitations	12
Discussion	21	Generalisability of the trial findings	12–13
Discussion	22	Interpretation consistent with results	11–13
Other information	23	Registration number and name of trial registry	1, Declarations
Other information	24	Where the full trial protocol can be accessed	ClinicalTrials.gov
Other information	25	Sources of funding and other support	Declarations
